# On the scent of sexual attraction

**DOI:** 10.1186/1741-7007-8-71

**Published:** 2010-06-03

**Authors:** Peter A Brennan

**Affiliations:** 1 Department of Physiology and Pharmacology, University of Bristol, Medical School, University Walk, Bristol BS8 1TD, UK

## Abstract

A study in the current issue of *BMC Biology *has identified a mouse major urinary protein as a pheromone that attracts female mice to male urine marks and induces a learned attraction to the volatile urinary odor of the producer. See research article http://www.biomedcentral.com/1741-7007/8/75

## 

It is more than 50 years since the term pheromone was proposed to label a category of chemical signals produced by one member of a species that elicit a definite response in another member of the same species. Since then, many pheromones have been identified in invertebrates, but the concept is less easily applied to vertebrates and especially to mammals. The problem is that robust pheromonal responses have proved difficult to identify in mammals. Indeed, mammalian behavioral responses are so strongly modulated by context and learning that this has led some investigators to question whether mammalian pheromones really exist [1].

## Sexual attraction

Work by Roberts and colleagues in this issue [2] has reinforced the concept of pheromonal communication in mammals by identifying a sexual attractant pheromone in male mouse urine. They used a robust behavioral assay in which receptive female mice are attracted to investigate and spend more time in proximity to urine from a sexually mature male, compared to female urine. It was already known that these attractant properties of male urine were associated with the non-volatile protein fraction [3]. By testing protein-containing sub-fractions of urine for their attractiveness, Roberts *et al*. [2] show that the attractive properties were present in a fraction containing a single major urinary protein (MUP) of 18,893 Da, which is consistently present in the urine of male, but not female, wild mice. They name this MUP darcin to distinguish it from the other MUPs present in the male urine that did not elicit attraction. This is not the first time that MUPs have been identified as potential pheromones. House mice have an unusual diversity of MUPs, much of which is due to the recent expansion of the class B central MUP genes, which have been adapted to signal the individual identity of urine marks in the context of territorial behavior [4]. MUPs have also been found to have a pheromonal effect in eliciting male aggression, although whether an individual MUP is responsible for this effect remains to be determined [5].

Like all MUPs, darcin is a member of the lipocalin family of ligand-binding proteins, and preferentially binds the volatile (*S*)-2-*sec*-butyl-4,5-dihydrothiazole (SBT), which itself has known pheromonal activity [6]. However, Roberts *et al*. showed that darcin's attractive properties remained when SBT had largely been lost, and that recombinant darcin was as attractive in the absence of normal mouse ligands. They thus confirmed that darcin itself was conferring on male urine the property of attracting females. It may seem counterintuitive that a non-volatile protein could function as a sexual attractant, as sexual attractants are more commonly thought to attract members of the opposite sex over considerable distances. However, many short-range sex pheromones also play an important role in advertising the presence and quality of a potential mate [7]. The attractant effect of darcin is a form of behavioral reinforcement, which occurs following direct contact. It increases the duration of investigation and reinforces a place preference for the vicinity of the stimulus. This attraction to urine marks will increase the effectiveness of other urinary constituents, such as the class B MUPs, in advertising male individuality and competitive ability. Interestingly, darcin is only found at trace levels in many inbred mouse strains of the Castle lineage, including many common laboratory strains such as BALB/c [8]. This is an important factor to consider when using these strains in studies of social and sexual behavior. But it also provides an opportunity to investigate the biological role of darcin by comparing behavioral responses and reproductive success among inbred strains that produce different levels of darcin.

## Pheromone-mediated odor learning

Roberts *et al*. [2] showed that in addition to being an attractant, darcin induced learning of the volatile odor of the urine mark. This is consistent with the behavioral reinforcement effect of darcin, which induces learning of contextual cues resulting in a place preference. The innate reinforcing properties of darcin are a feature of other mammalian pheromones, notably the rabbit mammary pheromone, 2-methylbut-2-enal. This volatile pheromone, sensed by the main olfactory system, elicits attraction to the mother's nipples and induces learning of volatile odorants with which it is associated [9]. These odors would normally be those of the mother and would act to reinforce the nipple-search response to the mammary pheromone during subsequent nursing episodes. In common with darcin, learning occurs during close physical contact between the mammary pheromone and the source of the learned odors. The reinforcement/learning role of short-range attractant pheromones is therefore distinct from classical long-range attractant pheromones, in which learning of contextual cues would be inappropriate.

As well as shedding fresh light on the nature of mammalian pheromones, and of mouse behavior, the identification of darcin provides a useful tool to trace the neural pathway from the vomeronasal organ to the neural circuits underlying behavioral reinforcement. The V2r class of vomeronasal sensory neurons mediates the action of all MUPs, but the darcin-responsive subpopulation must have a specific, labeled-line projection to brain reward circuits. Previous studies, using c-*fos *expression, point to the basolateral amygdala and nucleus accumbens as potential brain areas involved in the reinforcing effects of non-volatile urinary constituents [10]. But c-*fos *studies have to be interpreted with caution, and there are other sites in the amygdala by which main olfactory input could be linked with behavioral attraction. For instance, social odors predominantly activate the ventral region of the main olfactory bulb, which has a direct projection to the medial amygdala, overlapping with the vomeronasal projection from the accessory olfactory bulb [11]. Synaptic plasticity at this input to the medial amygdala potentially provides a direct link by which the main olfactory system could gain control over specific vomeronasal output circuits. It will therefore be interesting to investigate whether darcin can condition an attraction to non-social odors, as can occur with mammary pheromone odor conditioning in rabbits. If so, then this would point to darcin's role in inducing a more general contextual learning of stimuli associated with the urine mark, which would be more likely to involve the basolateral amygdala.

Darcin-mediated learning of main olfactory odors is not the only way in which mice learn to recognize individuals. It is well known that female mice learn to recognize their mate during a sensitive period around mating. This form of learning involves peptide individuality chemosignals related to the major histocompatibility complex, and is induced by the vaginocervical stimulation at mating. Hence females can recognize the identity of BALB/c males, in the context of the pregnancy-block effect, despite their lack of darcin production [12]. Mice therefore use different chemosensory cues and memory systems for individual recognition in different behavioral contexts.

## Darcin and mate choice

Mice readily learn to discriminate between volatile urine odors of individual mice, via the main olfactory system, on the basis of the MHC genotype of the producer [13,14]. However, urine odor is also likely to be influenced by environmental factors such as diet and infection status. The presence of darcin in urine marks thus enables the female to learn and constantly update the changeable volatile urine odor of the producer. This would enable the female to be able to quickly identify the producer of urine marks from a distance, without having to spend time in direct investigation of non-volatile individuality cues, such as class B MUPs. Interestingly, Roberts *et al*. [2] found that the addition of recombinant darcin to urine from BALB/c males, which normally contains only trace amounts of darcin, resulted in the females learning the volatile odor of the BALB/c urine containing darcin. But the learned attraction did not generalize to BALB/c urine without darcin. This lack of generalization of urine odors is important in maintaining the ability to discriminate and recognize individual males. However, the volatile odors released from a urine mark will change over time as the urine mark ages, and yet the attractant effect of darcin remains constant with age of the urine mark. This raises the question of whether the different composition of urinary volatiles given off by an aged urine mark generalizes to the odor of a fresh mark from the same male. It may be that only the freshest urine marks, which are the ones most likely to have been left by a competitive male, result in a strong attraction to the odor of the producer.

For female mice to enhance their reproductive success they need to choose a mate who will maximize the fitness of their offspring. One of the main ways they can achieve this is by assessing the competitive ability of males through their success in maintaining fresh urine marks over their territory and countermarking the marks of intruders. Females might be expected to encounter urine marks from a competitive male more frequently than marks of a less competitive male. Therefore, the darcin-conditioned attraction to urine marks of the most competitive male would be stronger than the attraction to the marks of a less competitive male. Urine marks would effectively compete for the attraction and attention of the females, and attraction to the most successful urine mark within a territory would increase her chances of attraction to, and sexual interaction with, the most competitive male. It will be interesting to see whether reinforcing effects are a common feature of pheromones used for territorial marking and sexual advertisement in other species.
